# Long non-coding RNA (lncRNA) five prime to Xist (FTX) promotes retinoblastoma progression by regulating the microRNA-320a/with-no-lysine kinases 1 (WNK1) axis

**DOI:** 10.1080/21655979.2021.1994718

**Published:** 2021-12-07

**Authors:** Xiaolei Wang, Yu Su, Chuangao Yin

**Affiliations:** a Department of Oncology, The Second Affiliated Hospital of Anhui Medical University, Hefei City, PR. China; b Department of Ophthalmology, Anhui Provincial Children’s Hospital, Hefei City, PR. China

**Keywords:** Retinoblastoma, FTX, miR-320a, WNK1

## Abstract

Long non-coding RNA (lncRNA) five prime to Xist (FTX) exerts important functions in human cancer, while its role in retinoblastoma (RB) remains unclear. This study aimed to investigate the role of FTX in RB. The expression levels of FTX were assessed by quantitative real-time polymerase chain reaction (qRT-PCR). Cell proliferation was evaluated by cell counting kit-8 (CCK-8), 5‐ethynyl‐2′‐deoxyuridine (EdU) staining and colony formation assays. Cell migration and invasion were detected by Transwell assay. The relationship among FTX, microRNA-320a (miR-320a) and with-no-lysine kinase 1 (WNK1) was also investigated. In the present study, we found that the expression levels of FTX were notably elevated in RB tissues and cancer cell lines. Overexpression of FTX exacerbated the aggressive phenotypes (cell proliferation, migration and invasion) of RB cells. Downregulation of miR-320a obviously attenuated the inhibitory effects of knockdown of FTX in RB malignant phenotypes, and knockdown of WNK1 also reversed the impacts of miR-320a inhibitor on malignant phenotypes. *In vivo* experiments further confirmed that knockdown of FTX efficiently prevents tumor growth *in vivo*. Our results revealed that FTX promoted RB progression by targeting the miR-320a/WNK1 axis (graphical abstract), suggesting that FTX might be a novel therapeutic target for RB.

## 1. Introduction

Retinoblastoma (RB) accounts for approximately 3% of all childhood cancers worldwide, and seriously endangers their visual functions and life [1]. RB always occurs along with leukocoria or strabismus, and the patients may suffer proptosis or hypopyon if untreated timely [2]. Although advances involved in the diagnosis have been made, the treatment of RB requires individualized therapies that depend on International Classification of Retinoblastoma (ICRB) staging [3]. Therefore, well understanding of the specific pathogenic mechanisms in RB is still necessary, and these attempts may provide novel therapeutic targets for this disease.

Long non-coding RNAs (lncRNAs) have emerged as the key regulators of ncRNAs with 200 nucleotides in length and lack protein encoding property [4]. It has been reported that lncRNAs exert essential regulatory functions in cancer progression by acting as the competing endogenous RNAs (ceRNAs) [5,6]. That is, lncRNAs can sponge microRNAs (miRNAs) to alter the expression of their target miRNAs in the development of human diseases [7,8]. Recently, an increasing number of lncRNAs closely participated in the occurrence and development of RB have been identified. For example, retinoblastoma-associated transcript-1 (RBAT1) accelerates the tumorigenesis of RB via targeting heterogeneous nuclear ribonucleoprotein L (HNRNPL) [9]. X-inactive-specific transcript (XIST) contributes to the proliferative rate and epithelial–mesenchymal transition (EMT) of RB cells by directly regulating miR-142-5p [10]. Five prime to xist (FTX) is a lncRNA that is encoded by the *FTX* gene [11] and was reported as an oncogene to participate in cancer progression in colorectal cancer [12], osteosarcoma [13], and renal cell carcinoma [14]. One previous study demonstrated that FTX might play a potential role in uveal melanoma (UM), also an intraocular malignant tumor [15]. Therefore, we speculated that FTX might participate in RB progression and this study was therefore carried out to investigate it.

In the present study, we investigated the potential oncogenic role of FTX in RB and the underlying mechanisms. Based on our data, we revealed for the first time that the expression levels of FTX were notably elevated in RB tissues and cancer cell lines. Overexpression of FTX exacerbated cell proliferative, migratory and invasive phenotypes of RB cells *in vitro*, while knockdown of FTX inhibited these malignant phenotypes. In addition, our data demonstrated that FTX played its regulatory roles in RB through targeting miR-320a to upregulate the expression of with-no-lysine kinase 1 (WNK1) (graphical abstract), providing a novel insight of FTX in RB.

## 2. Materials and methods

### 2.1. Tissue samples

A total of 30 pairs of retinoblastoma (RB) tissues and matched adjacent normal tissues were collected at Anhui Provincial Children’s Hospital from 2017 to 2020 through surgical excision. All participants provided written informed consent. None of the patients received relative treatment before the surgical excision.

### 2.2. Cell culture and transfection

RB cell lines (Y79, SO-RB50, and WERI-RB-1) and the normal retinal pigmented epithelial cell line ARPE-19 were purchased from the Cell Center of Chinese Academy of Sciences (Shanghai, China). RB cell lines were cultured with Dulbecco Modified Eagle Medium (DMEM, Life Technologies, Grand Island, NY, USA) supplemented with 10% fetal bovine serum (FBS, HyClone, Little Chalfont, UK). ARPE-19 cells were cultured with DMEM/F12 medium. To manipulate the levels of targets, the short hairpin RNA against FTX (sh-FTX), sh-WNK1, the nonsensical sequence (sh-NC), miR-320a mimics, inhibitor, as well as negative controls were purchased from RiboBio (Guangzhou, China). In addition, the overexpressing vectors pc-FTX (overexpression of FTX) were constructed by RiboBio. Next, 50 μM of sh-RNAs or 100 nM miR‐320a mimic/inhibitor or 100 ng overexpressing plasmids were transfected into RB cell lines using Lipofectamine 2000 (Invitrogen, Carlsbad, CA, USA). The sequences were listed as follows: Sh-FTX: 5ʹ-CTGCTACGACACTGAATTC-3ʹ; miR mimics: 5ʹ-AAAAGCUGGGUUGAGAGGGCGA-3ʹ; mimics NC: 5ʹ-UUCUCCGAACGUGUCACGUTT-3; miR inhibitor: 5ʹ-UCGCCCUCUCAACCCAGCUUUU-3ʹ; and inhibitor NC: 5ʹ-CAGUACUUUUGUGUAGUACAA-3.

### 2.3. Quantitative real-time polymerase chain reaction (qRT-PCR)

Total RNAs were isolated by TRIzol reagent (Thermo Fisher Scientific, USA) and RNA samples were reverse transcribed into complementary DNA (cDNA) using miScript Reverse Transcription Kit (Qiagen). Then PCR reactions were prepared using SYBR Green mixture (Qiagen) and PCRs were conducted on an ABI 7500 PCR system. The relative expression levels of the targets were calculated using the 2^−ΔΔCt^ method [16] with glyceraldehyde 3-phosphate dehydrogenase (GAPDH) and small nuclear RNA U6 as the internal controls. The sequences of primers were as follows: FTX, Forward: 5ʹ-GAATGTCCTTGTGAGGCAGTTG-3ʹ, Reverse: 5ʹ-TGGTCACTCACATGGATGATCTG-3ʹ; GAPDH Forward: 5ʹ-ATTCCATGGCACCGTCAAGGCTGA-3ʹ, Reverse: 5ʹ-TTCTCCATGGTGGTGAAGACGCCA-3ʹ; U6 Forward: 5ʹ-GCAGGAGGTCTTCACAGAGT-3ʹ, Reverse: 5ʹ-TCTAGAGGAGAAGCTGGGGT-3ʹ; miR-320a Forward: 5ʹ-GGGCTAAAAGCTGGGTTGA-3ʹ, Reverse: 5ʹ-CAGTGCGTGTCGTGGAGT-3ʹ.

### 2.4. Western blot

Total proteins were isolated using RIPA lysis buffer (Santa Cruz Biotechnology). Equivalent proteomic loads were separated by 12% sodium dodecyl sulfonate-polyacrylamide gel electrophoresis (SDS-PAGE), followed by transferring onto polyvinylidene fluoride membrane (PVDF, Millipore, Billerica, MA, USA). The membrane was consequently incubated against primary antibodies including anti-WNK1 (sc-8019; 1:500, Santa Cruz Biotechnology) or anti-GAPDH (sc-69,778; 1:10,000, Santa Cruz Biotechnology) at 4°C overnight. After further incubation with horseradish peroxidase (HRP)–conjugated secondary antibody (anti-IgG, sc-516,102; 1:5,000) for 1 h, the bands were observed using ECL kit and quantified by Image J software.

### 2.5. Cell proliferation

According to a previous study [17], cell aliquots were seeded into 96-well plates (2 × 10^4^ cells/well) and grown for different time periods. Consequently, cell aliquots were placed in incubation together with 10 ul of CCK-8 reagent (Cell Counting Kit-8, Dojindo Molecular Technologies, Gaithersburg, MD) for 4 h. Absorbance values at 450 nm were measured using a microplate reader.

### 2.6. 5‐ethynyl‐2′‐deoxyuridine (EdU) staining assay

The assay was performed using the 5‐ethynyl‐2′‐deoxyuridine (EdU) staining kit (Guangzhou RiboBio Co., Ltd., Guangdong, China) as previously described [18]. Briefly, 1 × 10^5^ RB cells were seeded into 24‐well plates and cultured overnight, followed by incubation with 50 μM EdU reagent for 3 h. Then DAPI solution was added to stain cell nucleus. The staining images were observed under a fluorescence microscope (Olympus, Tokyo, Japan), and the ratio of EdU positive cells to total cells was counted and calculated in five random fields.

### 2.7. RNA immunoprecipitation (RIP) assay

This assay was conducted using the Magna RIP RNA-Binding Protein Immunoprecipitation Kit (Millipore, USA) as previously described [19]. Briefly, the cultured cells were ruptured, followed by the post-lysis supernatants placed in incubation with magnetic beads conjugated human anti-Ago2 antibody or control mouse IgG (MilliporeTM, USA) at 4°C overnight. The co-precipitated RNAs were isolated, and qRT-PCR analysis was performed.

### 2.8. Bioinformatic prediction

The potential miRNA targets of FTX and downstream targets of miR-320a, as well as the putative binding sites were predicted by TargetScan (http://www.targetscan.org/), and StarBase 3.0 (http://starbase.sysu.edu.cn/), respectively [20].

### 2.9. Luciferase reporter assay

Luciferase reporter assay was performed as previously described [19]. The wild-type (WT) FTX (WT-FTX) or WNK 3ʹ-UTR (WT-WNK) and mutant of FTX (MUT-FTX) or WNK 3ʹ-UTR (MUT-WNK) containing miR-320 binding site were generated and cloned into psiCHECK™-2 luciferase reporter vector (Promina Corporation) to construct the recombinant luciferase vector FTX WT/MUT and WNK1 WT/MUT. Each plasmid was co-transfected with miR-320a mimics or miR-NC, targeting WERI-RB-1 cells using Lipofectamine 2000. After 48 h, the relative luciferase activity was detected using a Dual-Luciferase® Reporter Assay kit (Promega Corporation).

### 2.10. Tumor xenograft model

The *in vivo* animal model was constructed as previously described [21]. Approximately 1 × 10^7^ transfected WERI-RB-1 cells were subcutaneously implanted into the flanks of nude mice (BALB/c nude mice, n = 5). Tumor volume was evaluated every week for a total of 4 weeks using the equation: 0.52 × Width^2^ × Length. Finally, mice were euthanized by cervical dislocation, and tumors were removed and weighted. After that, the tumors were dissected, embedded in paraffin and sectioned at 5 μm, followed by Immunohistochemistry (IHC) staining as previously described [18]. After the incubation with anti-ki-67 antibody (1:500, ab16667, Abcam) at 4°C overnight and the secondary antibody anti-IgG (1:1,000, ab6721, Abcam) for 30 min, the tumor sections were observed under an ordinary fluorescence microscope.

### 2.11. Statistical analysis

The data were presented as the mean ± standard deviation (SD) using the SPSS v.19.0 software (IBM Corp., Armonk, NY, USA). The difference between two groups was tested by Student’s t test, and the comparisons between multiple groups were performed by one-way analysis of variance followed by Bonferroni’s post hoc test. Spearman’s correlation analysis was used to evaluate the correlation between the expression levels of FTX and miR-320a, as well as miR-320a and WNK1 in RB tissues. *P* < 0.05 was deemed to confer statistical significance.

## 3. Results

### 3.1. The expression levels of lncRNA prime to Xist (FTX) were elevated in RB tissues

**Figure 1.**
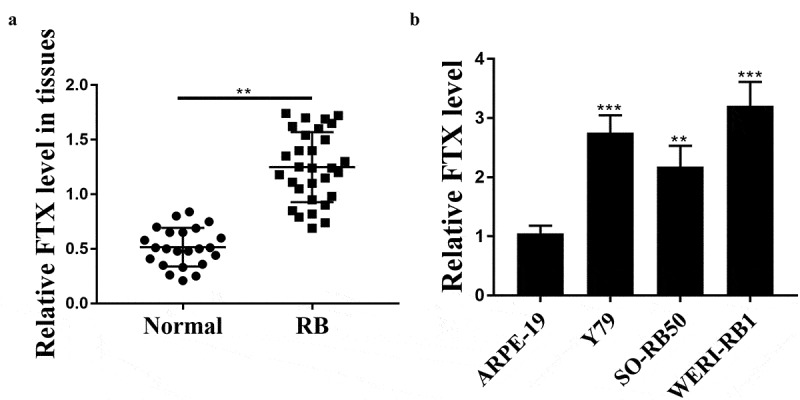
The expression of FTX in RB tissues and cancer cell lines. (a) The expression levels of FTX in RB tissues (n = 30) and RB cell lines (b) were assessed by qRT-PCR. ** *p* < 0.01, *** *p* < 0.001

To evaluate the role of FTX in RB, we detected its expression levels in RB tissues and cancer cell lines, and the results showed that the expression levels of FTX were notably elevated in RB tissues compared with that in adjacent normal tissues (*p* < 0.01, n = 30, Figure 1(a)). To confirm its upregulation, its expression was also detected in RB cell lines and the results confirmed its obvious upregulation in RB cell lines in comparison to that in ARPE-19 cells (Y79, *p* < 0.001; SO-RB50, *p* < 0.01; WER1-RB1, *p* < 0.001, Figure 1(b)). These results suggested that FTX may exert a potential role in RB progression.

### 3.2. Overexpression of FTX exacerbated the aggressive phenotypes of RB cells in vitro

**Figure 2.**
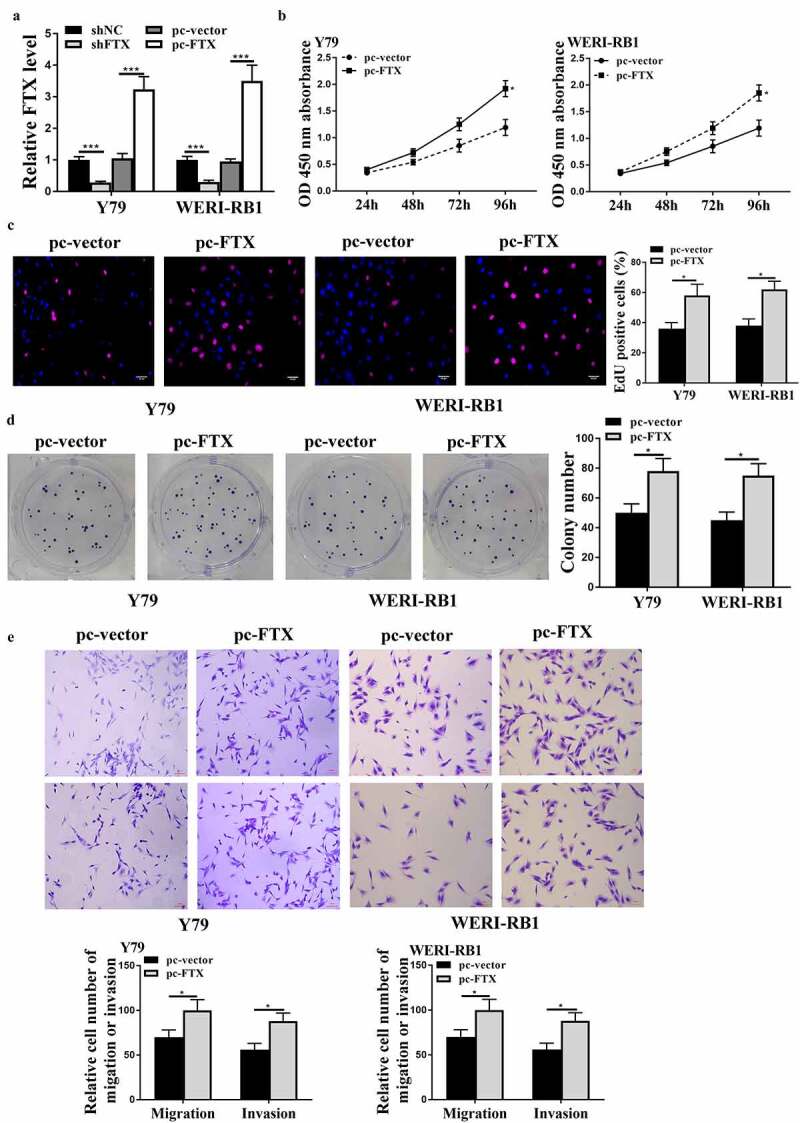
Overexpression of FTX exacerbated the malignant phenotypes of RB cells *in vitro*. Y79 and WER1-RB1 were transfected with pc-FTX (FTX overexpressing vector), sh-FTX and their corresponding negative controls. (a) The expression of FTX was evaluated by qRT-PCR. (b) The cell viabilities was determined by CCK-8 assay. (c and d) Cell proliferation was assessed by EdU staining assay (c) and colony formation assay (d). Scale bar = 40 μm. (e) Cell migratory and invasive capacities were evaluated by Transwell assay. Scale bar = 100 μm. * *p* < 0.05, *** *p* < 0.001

To investigate the function of FTX in RB, the pc-FTX (the overexpressing vector) and sh-FTX were transfected into Y79 and WER1-RB1 cells, and qRT-PCR results showed that the expression levels of FTX were increased in pc-FTX transfected cells and reduced in sh-FTX transfected cells compared with that in the control group (all *p* < 0.001, Figure 2(a)). Meanwhile, overexpression of FTX exacerbated cell proliferation of two cell lines compared with that in pc-vector control (all *p* < 0.05, Figure 2(b-d)). In addition, overexpression of FTX also increased the migratory and invasive abilities of two RB cell lines compared with that in pc-vector (all *p* < 0.05, Figure 2(e)). These findings suggested that FTX promoted RB progression.

### 3.3. FTX functioned as a competing endogenous RNA (ceRNA) of miR-320a

**Figure 3.**
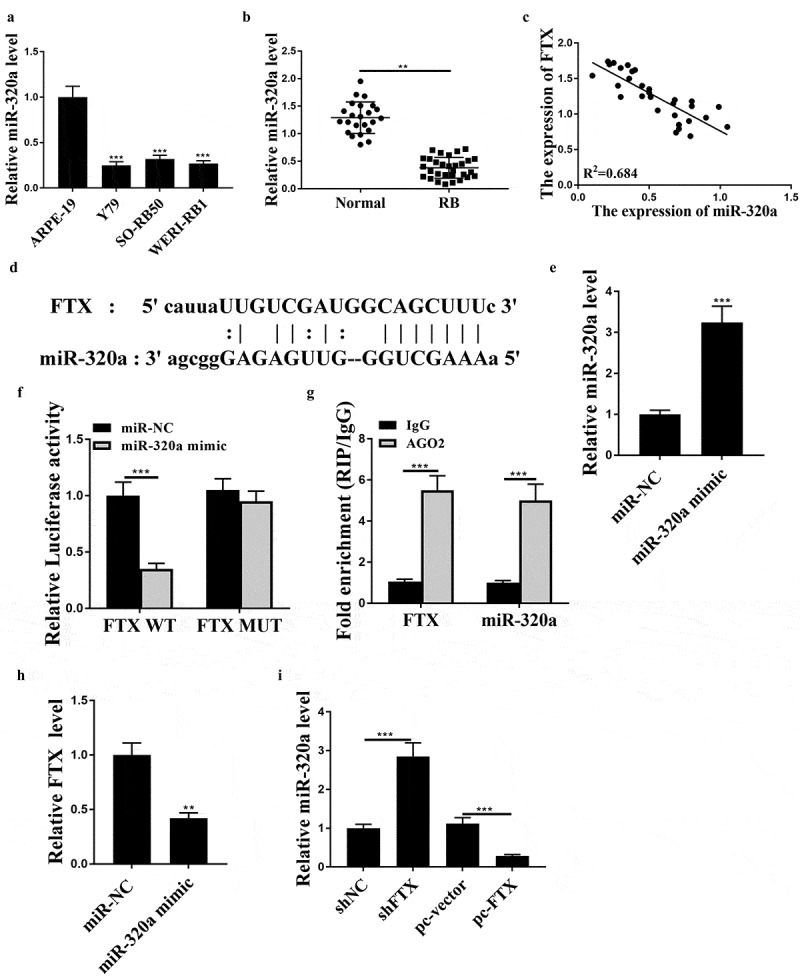
FTX functioned as a ceRNA of miR-320a. (a and b) The expression levels of miR-320a in RB cell lines (a) and RB tissues (b) were evaluated by qRT-PCR. (c) Pearson’s correlation analysis between the expression levels of FTX and miR-320a in RB tissues (n = 30). (d) The putative binding site between FTX and miR-320 was predicted by Starbase3.0. (e and f) WER1-RB1 cells were transfected with miR-32-a mimics or miR-NC. (e) The expression levels of miR-320a were evaluated by qRT-PCR. (f) Luciferase reporter assay. (g) The enrichment of miR-320a and FTX in WER1-RB1 cell supernatant was assessed by RIP assay using anti-Ago2 antibody. (h) WER1-RB1 cells were transfected with miR-320a mimics, and the expression levels of FTX were evaluated by qRT-PCR. (i) The expression levels of miR-320a were evaluated by qRT-PCR. ** *p* < 0.01, *** *p* < 0.001

To further investigate the mechanism of FTX, we assessed the expression of miR-320a in cancer cell lines and RB tissues. The results showed that the expression levels of miR-320a were notably reduced in RB cell lines (all *p* < 0.001) and RB tissues (*p* < 0.01) (Figure 3(a). Meanwhile, there was an obvious negative correlation between the expression levels of FTX and miR-320a in RB tissues (R^2^ = 0.684, Figure 3(c)). There was a putative binding site between these two molecules (Figure 3(d)), suggesting a direct interaction between them. Then miR-320a mimics was transfected into WER1-RB1 cells, and the transfection efficiency was determined by qRT-PCR (*p* < 0.001, Figure 3(e)). MiR-320a mimics obviously reduced the luciferase activity of FTX WT in WER1-RB1 cells (*p* < 0.001) but had no impact on FTX MUT (figure 3(f)). Also, both FTX and miR-320a were notably enriched in the Ago2 immune-precipitate compared with IgG group in WER1-RB1 cells (both *p* < 0.001, Figure 3(g)), further indicating the direct binding between FTX and miR-320a. MiR-320a mimics significantly increased the expression levels of FTX compared with miR-NC in WER1-RB1 cells (*p* < 0.01, Figure 3(h)). Moreover, knockdown of FTX increased the expression levels of miR-320a (*p* < 0.001), and overexpression of FTX reduced the expression levels of miR-320a (*p* < 0.001) in WER1-RB1 cells compared with that in corresponding negative controls (Figure 3(i)). These results suggested that FTX may participate in RB progression by sponging miR-320a as a ceRNA.

### 3.4. Knockdown of FTX inhibited RB progression through regulating miR-320a

**Figure 4.**
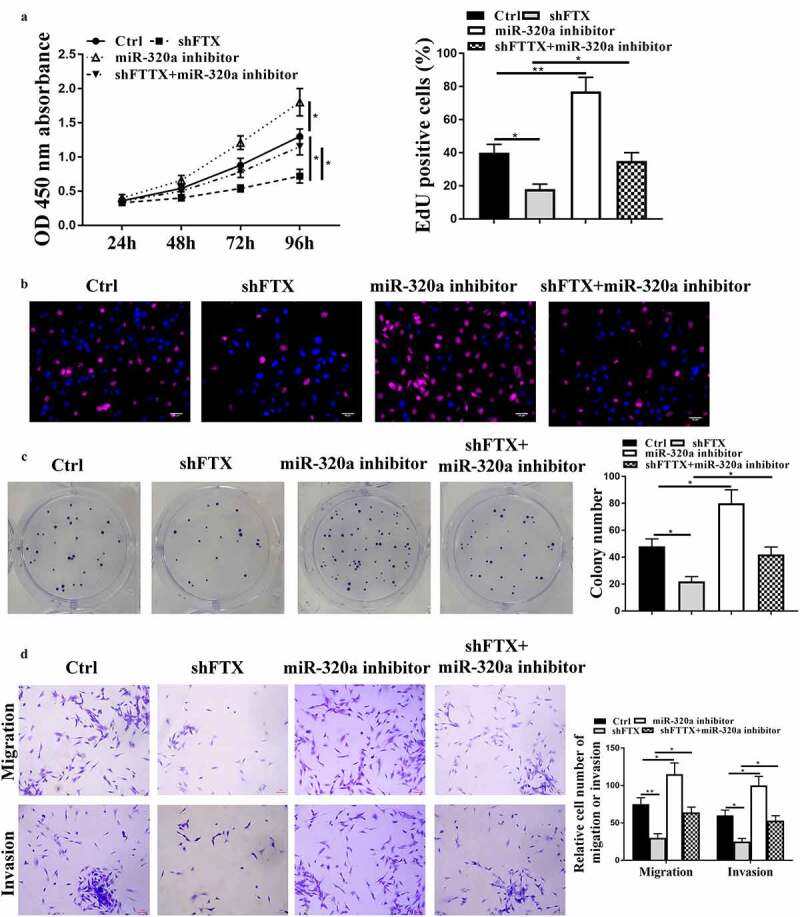
Knockdown of FTX inhibited RB progression through regulating miR-320a. WER1-RB1 cells were transfected with sh-FTX, miR-320a inhibitor, or co-transfected with sh-FTX and miR-320a inhibitor. (a) The cell viability was assessed by CCK-8 assay. (b and c) Cell proliferative rate was assessed by EdU staining assay (b) and colony formation assay (c). Scale bar = 40 μm. (d) Transwell assay. Scale bar = 100 μm. * *p* < 0.05, ** *p* < 0.01

To further determine whether FTX regulated RB progression through regulating miR-320a, the rescue experiments were conducted by transfecting miR-320a inhibitor and sh-FTX into RB cells. We found that knockdown of FTX significantly reduced cell viabilities (*p* < 0.05), EdU positive cells (*p* < 0.05) and colony formation (*p* < 0.05), while inhibited cell migratory (*p* < 0.01) and invasive capacities (*p* < 0.05) of WER1-RB1 cells compared with that in the control group, and downregulation of miR-320a inversely exacerbated these malignant phenotypes of WER1-RB1 cells (all *p* < 0.05). In addition, co-transfection obviously attenuated the inhibitory effects of knockdown of FTX in these malignant phenotypes of WER1-RB1 cells (all *p* < 0.05) (Figure 4(a-d)). These results suggested that miR-320a was closely involved in the regulation of FTX in RB.

### 3.5. With-no-lysine kinases 1 (WNK1) was a target of miR-320a

**Figure 5.**
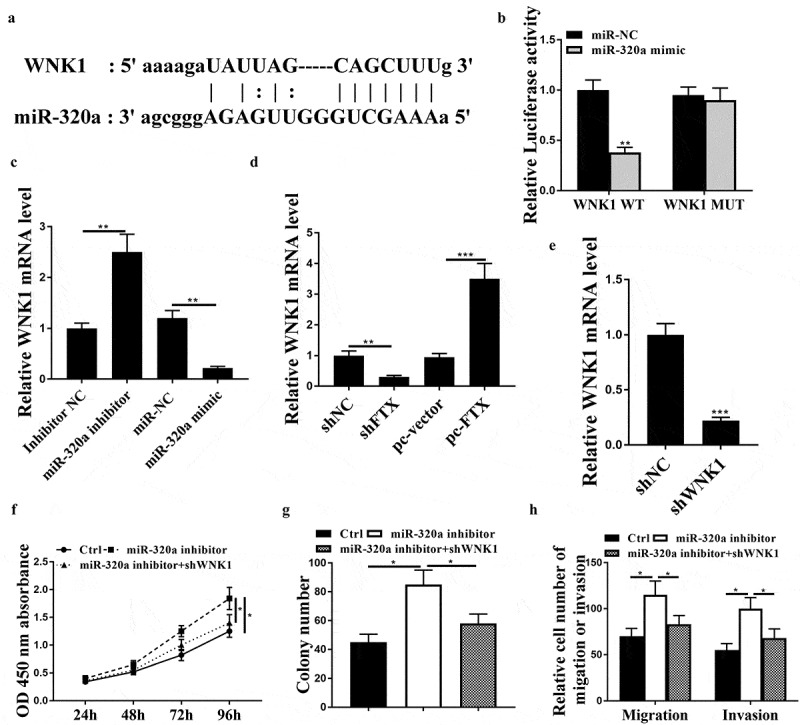
WNK1 was a target of miR-320. (a) Bioinformatic prediction of putative binding site between miR-320a and WNK1 by Targetscan. (b) Luciferase reporter assay. (c) WER1-RB1 cells were transfected with miR-320a mimics or inhibitor, and the expression levels of WNK1 were detected by qRT-PCR. (d) The expression levels of WNK1 were detected by qRT-PCR. (e) The expression levels of WNK1 were assessed by qRT-PCR. (f-h) WER1-RB1 cells were transfected with miR-320a inhibitor, or co-transfected with miR-320a inhibitor and sh-WNK1. (f) The cell viability was assessed by CCK-8 assay. (g) Cell proliferative rate was assessed by colony formation assay. (h) Cell migratory and invasive were evaluated by Transwell assay. * *p* < 0.05, ** *p* < 0.01, *** *p* < 0.001

Subsequently, we explored the regulatory mechanism of miR320a in RB, the potential targets of miR-320a were predicted by Targetscan and bioinformatic analysis indicated that WNK1 might be a target of miR-320a (Figure 5(a)). Luciferase reporter assay showed that miR-320a mimics significantly reduced the luciferase activity of WNK1 WT in WER1-RB1 cells (*p* < 0.01) but had no impact on WNK1 MUT (Figure 5(b)). MiR-320a inhibitor significantly increased the expression levels of WNK1 (*p* < 0.01), and miR-320a mimics reduced the expression levels of WNK1 compared with the corresponding controls in WER1-RB1 cells (*p* < 0.01, Figure 5(c)). Knockdown of FTX reduced the expression levels of WNK1 (*p* < 0.01), while overexpression of FTX increased the expression levels of WNK1 in WER1-RB1 cells (*p* < 0.001, Figure 5(d)). To confirm the regulatory axis of miR-320a and WNK1 in RB, sh-WNK1 was transfected into WER1-RB1 cells, and the expression of WNK1 was notably downregulated in sh-WNK1 transfected cells compared with sh-NC transfected cells (*p* < 0.001, Figure 5(e)). In addition, we found that co-transfection of miR-320a inhibitor and sh-WNK1 obviously attenuated the inhibitory effect of miR-320a inhibitor on cell viability, colony formation, migratory and invasive rates of WER1-RB1 cells (all *p* < 0.05, figure 5(f-h)). These data suggested that miR-320a participated in RB progression by targeting WNK1.

### 3.6. MiR-320a inhibitor attenuated the inhibitory effect of knockdown of FTX in tumor progression in vivo

**Figure 6.**
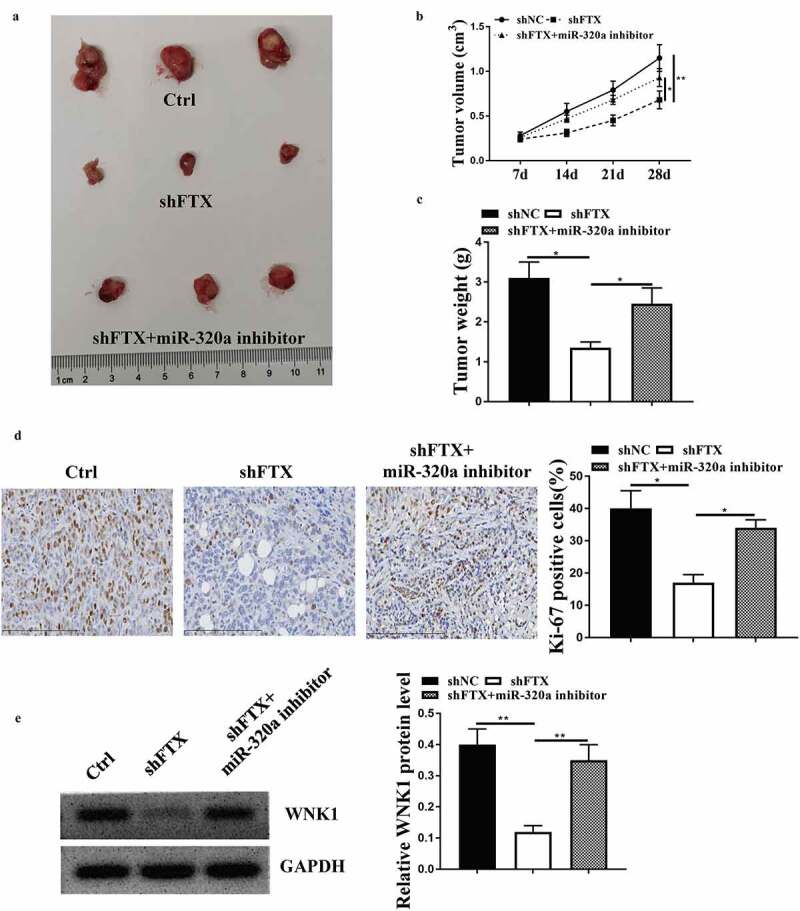
MiR-320a inhibitor attenuated the inhibitory effect of knockdown of FTX in tumor growth *in vivo*. (a) The representative tumor images. (b) Tumor volume. (c) Tumor weight. (d) Ki-67 staining assay. Scale bar = 50 μm. (e) Western blot for determining the expression of WNK1 in tumor. * *p* < 0.05, ** *p* < 0.01

To confirm the role of FTX *in vivo*, the *in vivo* animal model was established. As shown in Figure 6(a), knockdown of FTX obviously inhibited tumor growth, and co-injection of sh-FTX and miR-320a inhibitor obviously attenuated the inhibitory effects of knockdown of FTX on tumor growth (Figure 6(a)). Meanwhile, knockdown of FTX significantly inhibited tumor volume (*p* < 0.01) and weight (*p* < 0.05) compared with sh-NC, while co-injection attenuated the protective effects of knockdown of FTX (both *p* < 0.05, Figure 6(b). In addition, knockdown of FTX significantly reduced the number of EdU positive cells in tumor tissues (*p* < 0.05), while co-injection obviously attenuated its inhibitory effect (*p* < 0.05, Figure 6(d)). The effect of knockdown of FTX and miR-320a inhibitor on the expression of WNK1 was similar to that *in vitro* (both *p* < 0.01, Figure 6(e)). These results suggested that the FTX/miR-320a/WNK1 axis closely participated in RB progression.

## 4. Discussion

Recently, RB has attracted more attention due to its high common characteristic in childhood [22]. Increasing attempts have done in order to develop novel drug targets that might improve therapeutic effects for RB [23]. In this study, we investigated the function of FTX as well as its regulatory mechanism in RB progression. Our data revealed for the first time that the expression levels of FTX were elevated in RB and sharpen the malignant phenotypes of RB both *in vitro* and *in vivo* by modulating miR-320a and WNK1, suggesting that FTX might be a novel therapeutic target in this disease.

It has been reported that FTX was commonly upregulated in several human cancers and closely participate in tumor behaviors to promote cancer progression including hepatocellular carcinoma [24], lung adenocarcinoma [25], and gastric cancer [26]. Considering the wide impact of FTX in human cancer, we speculated that FTX might also exert similar expression pattern and functions in RB development. Our study confirmed the upregulation of FTX in RB, and high expression levels of FTX were positively related to RB development, while knockdown of FTX suppressed RB progression. These data verified our hypothesis. In addition, one previous study found that FTX was also associated with the progression of uveal melanoma (UM), which is another intraocular malignant tumor [15]. Hence, FTX might also play a potential role in other intraocular malignant tumors, and we only focused on its role in RB. Based on the results in this study, we confirmed that FTX played an oncogenic role in RB.

Increasing evidence has demonstrated that lncRNAs and miRNAs were often interacted in cancer biology [27]. FTX also interacts with different miRNAs in different types of human cancer, such as miR-590-5p in colorectal cancer [28], miR-214-5p in osteosarcoma [29], and miR-335-5p in lung adenocarcinoma [30]. In this study, we predicted the potential miRNA targets of FTX using Starbase 3.0, and found that FTX might bind to miR-320a and sponge it. The RIP and luciferase reporter assays further determined their direct interaction. MiR-320a was downregulated in several tumor tissues and was found to inhibit the malignant phenotypes in some malignancies such as melanoma [31], gastric cancer [32], and glioma [33]. In the present study, a negative correlation between the expression levels of FTX and miR-320a in RB tissues was observed. MiR-320a inhibitor exacerbated the progression of RB cells and attenuated the inhibitory impacts of knockdown of FTX in RB progression. Despite the downregulation of miR-320a in RB, and inhibition of miR-320a inhibited the aggressive phenotypes of RB cells in a previous study [34], we actually confirmed that miR-320a inhibited the malignant phenotypes and acted as a tumor suppressor in RB.

MiRNAs could regulate the expression of downstream genes by binding to their 3ʹ-UTR and inhibiting their expression at post-transcriptional level [35]. Here, we found that WNK1 was a target of miR-320a. Previous studies reported the high expression levels of WNK1 in RB and contributed to RB progression and even was considered as probable therapeutic target for RB [36]. In this study, the rescue experiments revealed that knockdown of WNK1 obviously improved RB progression caused by miR-320 inhibitor. The expression of WNK1 conformed to the regulatory relationship of FTX and miR-320a. Increasing reports have identified various downstream genes of miR-320a including programmed cell death protein 1 (PD1) in malignant mesothelioma [37], interleukin 4 in preeclampsia [38], microtubule-associated protein 9 (MAP9) in postmenopausal osteoporosis [39], and Rab protein 14 (RAB14) in gastric cancer [40]. In future studies, more attention should be paid on these known target genes identified in previous studies in RB progression.

Moreover, the *in vivo* animal model further confirmed the oncogenic role of FTX in RB, as the regulatory network of FTX/miR-320a/WNK1. However, there was a limitation in this study, whether manipulating the expression levels of WNK1 *in vivo* affect the function of FTX or miR-320a needed to be determined in future.

## 5. Conclusion

In summary, our results demonstrated that knockdown of FTX could obviously inhibit RB development through regulating miR-320a and WNK1 axis, providing that FTX was a potential diagnostic and therapeutic marker in this disease.

## Research highlights

- FTX was significantly upregulated in RB tissues.
- Knockdown of FTX could inhibit the RB progression both *in vitro* and *in vivo*.
- The miR-320a/WNK1 axis participated in the regulation of FTX in RB development.
